# Clinical investigation of back disorders in horses: A retrospective study (2002-2017)

**DOI:** 10.14202/vetworld.2019.377-381

**Published:** 2019-03-12

**Authors:** Abubakar Musa Mayaki, Abdul Razak Intan-Shameha, Mohd Adzahan Noraniza, Mazlan Mazlina, Lawan Adamu, Rasedee Abdullah

**Affiliations:** 1 Department of Veterinary Preclinical Sciences, Faculty of Veterinary Medicine, Universiti Putra Malaysia, 43400 Serdang, Selangor, Malaysia; 2 Department of Veterinary Medicine, Usmanu Danfodiyo University, P.M.B. 2346, City Campus Complex, Sokoto, Nigeria; 3 Department of Farm and Exotic Animal Medicine and Surgery, Faculty of Veterinary Medicine, Universiti Putra Malaysia, 43400 Serdang, Selangor, Malaysia; 4 Department of Veterinary Pathology and Microbiology, Faculty of Veterinary Medicine, Universiti Putra Malaysia, 43400 Serdang, Selangor, Malaysia; 5 Department of Veterinary Clinical Studies, Faculty of Veterinary Medicine, Universiti Putra Malaysia, 43400 Serdang, Selangor, Malaysia; 6 Department of Veterinary Laboratory Diagnosis, Faculty of Veterinary Medicine, Universiti Putra Malaysia, 43400 Serdang, Selangor, Malaysia

**Keywords:** back disorder, diagnosis, horses, Malaysia, management

## Abstract

**Background and Aim:**

Back disorder is an ailment that often affects athletic and riding horses. Despite the rapidly growing equine athletic and equestrian activities, there is no documentation on the nature of equine back disorder (EBD) in Malaysian horses. The purpose of this study was to characterize EBD cases presented to University Veterinary Hospital, Universiti Putra Malaysia, between 2002 and 2017.

**Materials and Methods:**

The compilation of data was based on signalment, case history, duration of clinical signs, anatomical location of the pain, method of diagnosis, type of EBD, treatment, and outcome. The diagnosis of EBD was based on a history of poor performance, clinical examination findings, radiography, and, where applicable, necropsy.

**Results:**

A total of 181 diagnosed cases of EBDs were identified. The age of horses ranged from 5 to 22 years. The EBD cases were more prevalent in male than female horses and predominantly in geldings (60.77%). Thoroughbred, Arab, Polo pony, and Warmblood also recorded the most EBD cases among breeds. The discipline of horses tended to influence the development of EBDs, with patrolling horses recording the highest frequency. Most EBD cases were of the primary type (92.27%), with the main causes being soft-tissue lesions (57.48%), vertebral lesions (18.56%), tack-associated problems (16.77%), and neurological lesions (7.19%). The common treatments employed were administration of nonsteroidal anti-inflammatory agents, 1 to 3-month rest, warm and cold compression therapy, massage therapy, exercise adjustment, as well as correction of ill-saddle fit.

**Conclusion:**

Most EBDs in this study were associated with soft-tissue lesions. Among vertebral lesions, kissing spines were the most common cause of EBDs in horses in Malaysia.

## Introduction

Equine back disorder (EBD) is an ailment often inflicting athletic and riding horses, and the number of cases is on the increase [1]. The condition is usually caused by disorders of any of the spinal structures, and the main signs are back pain and loss or decrease in performance. The range of reported prevalence of EBD is wide, from 1 to 94% [2]. There is no age or sex predisposition for EDB; however, more cases were seen in horses aged 5 years and older and in Thoroughbreds, Warmbloods, Quarter horses, and Arabs than other breeds [3-5]. EBD can be classified as primary or secondary disorders. Primary EBDs are associated with lesions in any of the spinal structures such as those caused by muscle, ligament, osseous/vertebral, and nerve injuries [6]. Secondary EBDs are the result of strain from axial skeleton lesions, most commonly in lameness of either the forelimbs or hindlimbs.

In veterinary practice, the precise understanding of how animals’ express pain is an important determinant for adequate diagnosis and effective pain management [7-9]. The evaluation of back pain and diagnosis of EBD are very difficult and challenging because of the multistructural arrangement of the spine, difficulty in palpating huge and deeper structures, the type of back problem, occasional spontaneous recovery, multiple spinal lesions, lameness associated with back pain, and false interpretation of the response to grooming or touch as back pain [6,10]. Many equine clinicians rely on the subjective assessment of case history including injuries, abnormal physical functions, and poor performance and physical examinations including posture and conformational defect, pain response, muscular and vertebrae symmetry, and response to local anesthesia [6,11]. Others use imaging techniques such as radiography, ultrasonography, scintigraphy, and thermography to determine the structures involved in the disorder for better diagnosis [12,13]. Radiography has been the most popular imaging modality for osseous lesions; however, scintigraphy, a technique using radioactive tracers and gamma camera for the detection of bone diseases and ultrasonography for soft tissues injuries, seems to provide better means of diagnosis [14,15]. The challenges associated with the diagnosis of EBDs are also contributed by the failure to institute the right therapy or management plan. Treatment of the condition is often to relieve the pain with nonsteroidal anti-inflammatory agents. However, unresponsiveness to treatment and recurrence of the condition have called for alternative therapeutic plans including surgery as in cases of kissing spines [16], mesotherapy, hydrotherapy, chiropractic therapy [2], magnetic therapy, shock-wave therapy, acupuncture [17], and massage and stretching exercises [18]. In EBD, particularly kissing spines, surgical treatment is beneficial because it increases the recovery rate of horses and allows for their early return to normal activities [16,18,19]. Recovery in EBD can be further facilitated with exercise therapy to improve spinal musculoskeletal functions [3,20].

In Malaysia, cases of EBDs are becoming more prevalent, primarily due to the rapid growth in equine sport. Thus, the objective of this study was to determine the characteristics of EBD and review the management of these cases in Malaysia.

## Materials and Methods

### Ethical approval

This study was approved by the Institutional Animal Care and Use Committee (IACUC), Universiti Putra Malaysia, Selangor, Malaysia (No. R016/2018).

### Case selection criteria

The study was conducted on 181 equine cases presented to the University Veterinary Hospital, University Putra Malaysia (UVH-UPM), between the years 2002 and 2017. The case selection criteria used for this study include cases presented with the complaint of back pain and those with clinical signs consistent with back problem, such as marked response to palpation and manipulation, muscle stiffness, and ill-fitting saddle lesions.

### Demographic and methods of diagnosis

The age, sex, breed, horse activity, case history, duration of signs at presentation, anatomical location of pain, and treatment and outcomes were recorded. Horses with a history of poor performance, muscle soreness, stiffness or atrophy, evidence of fractured dorsal spinous process (DSP) or other vertebrae, overriding DSPs, and ill-fitted saddle were diagnosed with EBD. Radiography (26 cases) and, where applicable, necropsy (10 cases) were used to confirm the anatomical location of lesions causing back pain.

### Distribution and classification of EBDs

The frequency of EBD according to the breed and discipline of the horses was recorded. Based on the history and complaints, the EBD cases were also categorized as acute, if the signs were seen for less than a week; chronic, if more than a week; and recurrent if the cases recurred or showed no improvement after treatment. The EBDs were further classified into primary and secondary types based on the structures involved. Primary EBDs were further sub classed into vertebral, soft tissue, and neurological lesions and tack-associated problems, based on the structures involved in the development of the abnormality. The lateral view of the thoracic and lumbar radiographs of the DSPs was used to confirm kissing spines, and the diagnosis was made if there is loss or narrowing of interspinous spacing, two or more spinous processes either touching or overlapping, and complete fusion of the spinous processes (Table-1) [21].

**Table-1:** Radiological classification of kissing spines [21].

Grade	Description
0	Normal spacing between the spinous processes
1	Narrowing of the interspinous space
2	Densification of margins of the DSPs
3	Bone lysis adjacent to margins of the DSPs
4	Severe remodeling of the DSPs
5	Complete fusion with the adjacent spinous process

DSPs=Dorsal spinous processes

### Statistical analysis

Data generated from the clinical records were analyzed using MedCalc Statistical Software version 18.5 (MedCalc Software, Ostend, Belgium; http://www.medcalc.org; 2018) and summarized using descriptive statistics. The frequency, proportion, and confidence interval of EBD cases were analyzed according to gender, breed, horse activities, and type of back disorder.

## Results

### Signalments

**Figure-1:**
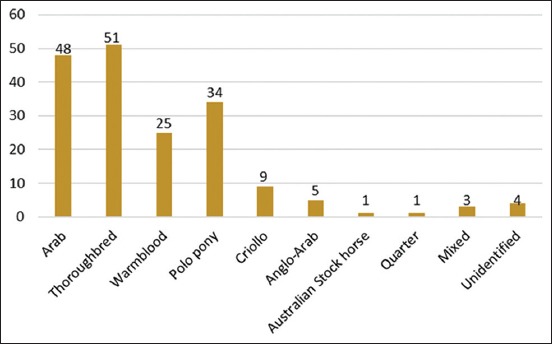
Breed distribution of the equine back disorders.

**Figure-2:**
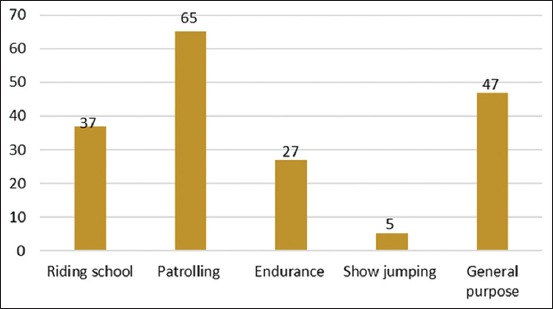
The classification of equine back disorder cases based on horse disciplines.

A total of 181 horses presented to the UVH-UPM were identified to suffer from EBD. The age distribution of horses with back disorder was 5–22 years, with the 9-year-olds showing the highest frequency. Among the cases, 67.40% were males: Gelding (60.77%), stallion (6.63%), and mares (32.60%). The EBD cases were highest among Thoroughbreds (28.18%), Arabs (26.52%), polo ponies (18.78%), and Warmblood horses (13.81%) (Figure-1). Based on the horse discipline (Figure-2), the frequency of back disorder cases was highest among patrol (35.91%), followed in order by riding school (20.44%), endurance (14.92%), and show jumping horses. Forty-seven (25.97%) horses involved in more than one discipline were grouped as general-purpose horses. Among the general-purpose horses, 72.3% were endurance and patrol, 14.9% patrol and show jumping, 6.4% endurance and riding school, and 6.4% show jumping, riding school, and dressage horses.

### Diagnosis and classification of EBDs

Based on the complaints and history, the frequency of acute EBDs was 41.44%, chronic EBDs was 51.93% (mostly due to neurologic lesions), and recurrent EBDs was 6.63%. Of all back disorder cases, 92.27% were primary EBDs and 7.73% were EBDs concurrent with lameness (i.e., secondary EBDs) involving forelimbs (2.21%) and hindlimbs (5.52%). The primary EBDs were due to vertebral lesions, soft-tissue lesions, neurological lesions, and tack-associated problems (Table-2). The most common site of soft-tissue lesions was epaxial muscles, particularly the longissimus dorsi muscle. There were 31 cases of vertebral lesions, of which 80.65% were due to overriding DSPs (kissing spines), 9.68% due to spondylosis, 6.45% due to osteoarthritis, and 3.22% due to fractured dorsal spinal processes. The pain perception in most of the vertebral lesion cases was in the thoraco-lumbar (T6-L5) region of the spine except for three cases, which was in the lumbosacral region.

**Table-2:** Types and distribution of primary EBD.

Primary EBD type	Diagnosis	Total cases number (%)	95% CI (%)
Soft tissue lesions	Muscle soreness and swollen	96 (57.48%)	49.91-64.74
Vertebral lesions	Overriding DSPs, spondylosis fracture of dorsal spinal processes, osteoarthritis	31 (18.56)	13.14-25.47
Neurological lesions	Spinal injury or compression	12 (7.19%)	4.16-12.51
Tack-associated problems	Saddle sore ill-fitting saddle	28 (16.77%)	11.61-23.5

EBD=Equine back disorder, CI=Confidence interval

**Figure-3:**
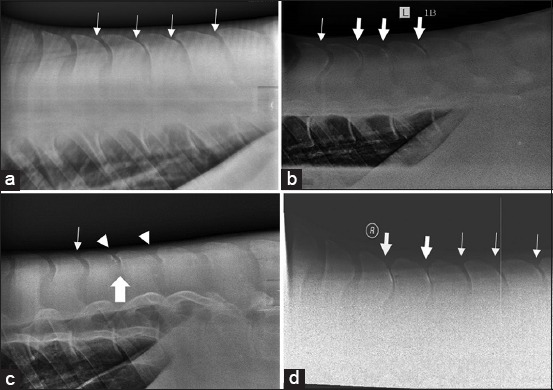
Lateral views of the caudal thoracic and lumbar dorsal spinous processes (DSPs) of horses with back pain. (a) Narrow spacing between DSPs (thin arrow) and bone lysis (b) narrow spacing between DSPs (thin arrow) and kissing of DSPs (thick arrow). (c) Radiodense (bright white) area is indicative of bone surface remodeling (arrowhead) and bone growth (big arrow). (d) Narrow spacing between DSPs (thin arrow) and kissing of DSPs (thick arrow).

Radiography was used to confirm 26 cases with kissing spines, whereas necropsy confirmed 10 cases with kissing spines, dorsal spinal processes fractures, and spinal injury or compression. Kissing spine lesions ranged from narrowing of interspinous spacing to the development of bony growth at the tip of the spinous process that gradually caused narrowing of the interspinous space. In these cases, two or more touching spinous process involved 4^th^ and 8^th^ or 9^th^ vertebrae (Figure-3). The compressive spinomalacic lesions characterized degenerative changes in the gray and part of white matter, dilatation of myelin sheaths, axon loss, and astrogliosis.

### Management and outcome of the conditions

In general, conservative treatments include nonsteroidal anti-inflammatory agents, especially phenylbutazone and flunixin meglumine, 1 to 3-month rest, warm and cold compression therapy, massage therapies, modification of exercise, as well as correction of ill-fitted saddle. There were complete follow-up records for 157 horses, out of which 15.29% showed spontaneous recovery without any treatment. Complete recovery was recorded following treatment in 61.65% of cases. These cases were mostly due to soft-tissue lesions and tack-associated problems. No improvement was observed in 38.35% of cases with two horses dead and eight euthanized. The pathological findings in these cases were kissing spines, osteoarthritis, and compressive myelopathic lesions.

## Discussion

Back pain is an ongoing welfare issue among working horses. In athletic horses, back pain is the major cause of loss of performance. It is suggested that the incidence of back problems is dependent on the type of injury and the back structure involved [6,22]. In addition, long-backed horse is more susceptible to back weakness than short-backed horses [4]. This presumably is due to long-backed horses tending to experience more soft-tissue injuries, whereas short-backed horses are more inclined to develop osseous lesions. However, our study, like several previous reports [5,23], showed that there is no obvious difference in the incidence of EBD between short-backed and long-backed horses. At this point, although there is no reported association between breed and predisposition in EBDs, the majority of horses presented to the veterinary hospital were Arabs, Thoroughbreds, Warmblood horses, and polo ponies. The high frequency of EBDs in these breeds, as shown by other studies [5,23], may reflect breed preponderance rather than a predisposition to back problems. The age range of horses (5-22 years) with EBDs observed in the study is similar to that report by other studies [4,5]. The number of geldings was higher than that of stallion or mares; subsequently, more cases of EBD were seen in these horses.

Most horses with EBD in this study were patrol horses followed by general and riding school horses, with show jumping horse the least in number. There was greater incidence of EBD with thoracolumbar and sacroiliac joint pains among show jumping and dressage horses [4,24]. However, it is difficult to conclude from our study whether specific physical activities influence the incidence of back problems because of the unequal distribution of horses representing various disciplines in Malaysia. According to horse-owners, the duration of cases before presentation varied from less than a week to several weeks. Assessment of the cases showed that there were more primary than secondary EBDs. Although it was reported by other studies that EBD is associated with a high prevalence of lameness [25], our study showed that, in Malaysian horses, cases of back pain with concurrent lameness were low in number, more so since stiffness and poor performance in these horses were signs of back pain rather than lameness.

Kissing spines has been considered the most common osseous lesions contributing to poor performance in athletic horses because of its association with thoracolumbar pain. From clinical signs and radiographic evidence, the incidence of kissing spines among horses with back pain in our study is most commonly under the saddle (Figure-3), which is similar to that reported earlier [3,5,15]. Similar to a previous study [24], kissing spine is a common vertebral lesion in horses, and those involving more than three vertebrae were also observed in back pain associated with poor performance. Several studies report similar risks of developing back disorders’ potential and kissing spine syndrome [4,5,26]. Because kissing spines in equine thoracolumbar pain markedly affect performance and the condition is not responsive to conservative treatments, there is a need to explore and discover more effective alternative treatments [16]. Other vertebral problems associated with EBD include intervertebral articulation [19] and disc herniation causing spinal compression, peripheral neuropathy, and nerve pinching. These conditions are similar to those seen in human and dogs. However, this was evident at necropsy, where compressive spinomalacia was observed in some case of chronic back disorders.

The longissimus muscle and supraspinous and dorsal sacroiliac ligaments are the most common soft-tissue lesions in EBDs. Thus, the palpation and manipulation method used in this study to determine EBD is acceptable, although imaging techniques such as ultrasonography would provide more objective diagnoses [14]. Since longissimus dorsi muscle’s action is to control the movement of the back either during bending of the spine or extending the back, injuries to this muscle commonly occur during riding. Saddle sore and ill-fitting saddle are the tack-associated often-causing EBDs [27].

## Conclusion

Most EBDs in this study were due to soft-tissue lesions. Among vertebral lesions, kissing spines were the most common cause of EBDs in horses in Malaysia.

## Authors’ Contributions

The research was conceived by AMM and ARIS and designed by AMM, ARIS, MAN, and RA. The data were collected by AMM and analyzed by AMM and LA. The manuscript was written by AMM and ARIS and revised by MAN, MM, LA, and RA. All authors have read and approved the final manuscript.
